# Effect of NET-1 siRNA conjugated sub-micron bubble complex combined with low-frequency ultrasound exposure in gene transfection

**DOI:** 10.18632/oncotarget.23646

**Published:** 2017-12-23

**Authors:** Bolin Wu, Xitian Liang, Hui Jing, Xue Han, Yixin Sun, Cunli Guo, Ying Liu, Wen Cheng

**Affiliations:** ^1^ Department of Ultrasound, Harbin Medical University Cancer Hospital, Nangang, Harbin, 150081, Heilongjiang Province, China

**Keywords:** sub-micron bubbles, low-frequency ultrasound, NET-1 siRNA, hepatocellular carcinoma, gene silencing

## Abstract

The present study evaluated the effect of NET-1 siRNA-conjugated sub-micron bubble (SMB) complexes combined with low-frequency ultrasound exposure in gene transfection. The *NET-1* gene was highly expressed level in SMMC-7721 human hepatocellular carcinoma cell line. The cells were divided into seven groups and treated with different conditions. The groups with or without low-frequency ultrasound exposure, groups of adherent cells, and suspension cells were separated. The NET-1 siRNA-conjugated SMB complexes were made in the laboratory and tested by Zetasizer Nano ZS90 analyzer. Flow cytometry was used to estimate the transfection efficiency and cellular apoptosis. Western blot and quantitative real-time polymerase chain reaction (qPCR) were used for the estimation of the protein and mRNA expressions, respectively. Transwell analysis determined the migration and invasion capacities of the tumor cells. The results did not show any difference in the transfection efficiency between adherent and suspension cells. However, the NET-1 siRNA-SMB complexes combined with low-frequency ultrasound exposure could enhance the gene transfection effectively. In summary, the NET-1 siRNA-SMB complexes appeared to be promising gene vehicle.

## INTRODUCTION

Currently, cancer cannot be cured using the traditional treatments such as surgery, radiotherapy, and chemotherapy. However, many patients with liver cancer manifest early vascular invasion [1]. Although hepatocellular carcinoma (HCC) is the sixth most common cancer and the third most common cause of cancer-related deaths worldwide [2, 3], standard chemotherapy shows a poor treatment efficacy [4]. Therefore, gene silencing therapy has been developed as a novel therapeutic strategy in recent years. Gene silencing and delivery have been demonstrated as promising methods for the treatment of cancer [5, 6]. However, the low transfection efficiency to specific tissues or organs impeded its wide clinical applications. Nevertheless, low-frequency ultrasound (LFUS) could enhance the transient permeability of plasma membranes to facilitate the uptake [7].

LFUS could improve the efficiency of gene delivery into tissues and cells, thereby supporting the acoustic cavitation [8] by creating holes of approximately 300 nm on the cytomembranes or transient pores in vascular endothelial cells. On the other hand, microbubbles (MBs) are known as the cavitation nuclei that would burst during the exposure to LFUS [9]. The acoustic cavitation could be enhanced further in combination with MBs [10].

Microbubbles, such as SonoVue, have been used as ultrasound contrast agents in medical ultrasound imaging for several years. Recently, MBs has been regarded as a vehicle for gene silencing owing to their visibility in the target organ [11–13]. However, a fatal disadvantage of microbubbles is their limited applicability in tumor therapy, especially, their large size prevents the penetrate through the endothelial gaps of vasa vasorum [14]. Consequently, the sub-micron bubbles (SMBs) were found to be optimal for gene delivery [15] as they could passage the endothelial gaps and the interstitial spaces of tumor tissues unobstructed [16]. Furthermore, the siRNA could be conjugated with SMBs, such that the shell of SMBs could protect the siRNA-SMB complexes against nucleases [17, 18].

Serru *et al.* identified the neuroepithelial transforming protein 1 (*NET-1*) gene [19]. It belongs to the tetraspan superfamily (TM4SF), which is highly expressed in HCC [20, 21]. The full-length NET-1 mRNA is 1297 bp encoding an open reading frame of 241 amino acids [22]. Therefore, the *NET-1* gene might be an effective target for gene therapy of HCC.

Herein, we prepared the NET-1 siRNA-conjugated SMB complexes and hypothesized that these complexes could facilitate the transfection of siRNA when combined with LFUS exposure. The NET-1 siRNA was transfected into the suspension and adherent SMMC-7721 cells, independently in order to assess the transfection efficiency using a variety of experimental methods.

## RESULTS

### Fabrication and characterization of siRNA-SMB complexes

**Figure 1:**
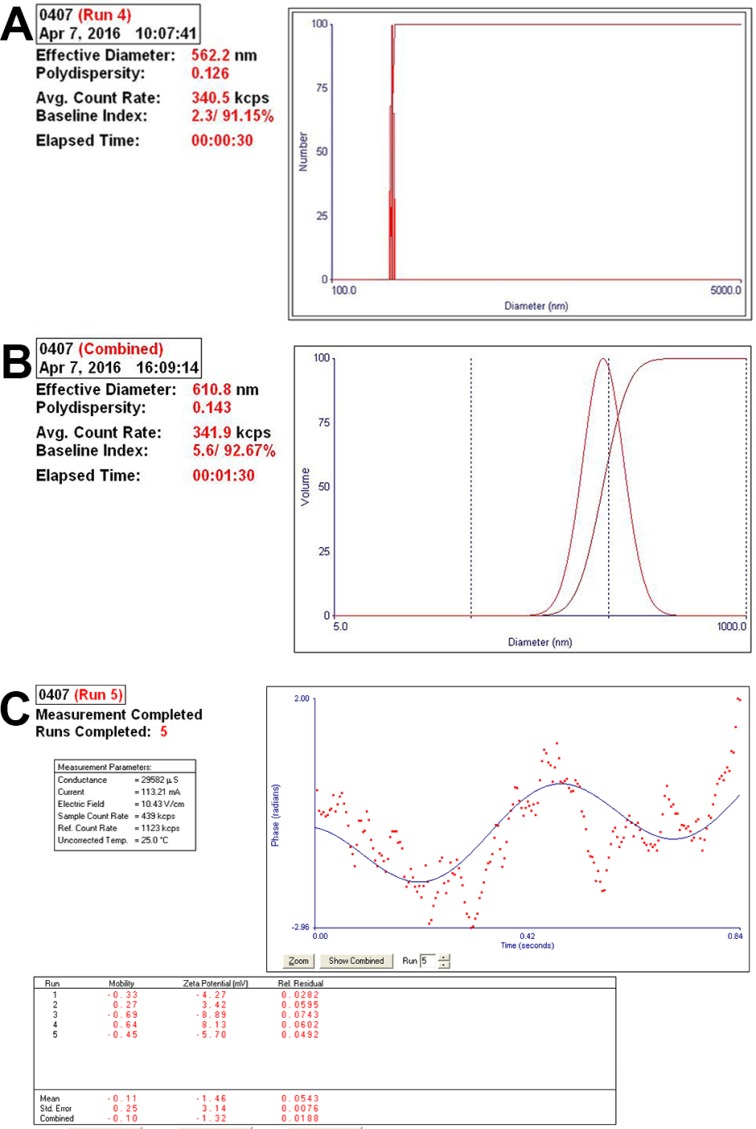
Analysis of the particle size and diameter of complexes (**A**) The complexes showed small diameters and optimal polydispersity. (**B**) The effective diameter was 610.8 ± 26.3 nm, and hence, the complexes could penetrate the interval of vascular endothelial cells to deliver the siRNA into cells. (**C**) The zeta potential value of the complexes was –1.46 ± 3.14 mV.

**Figure 2:**
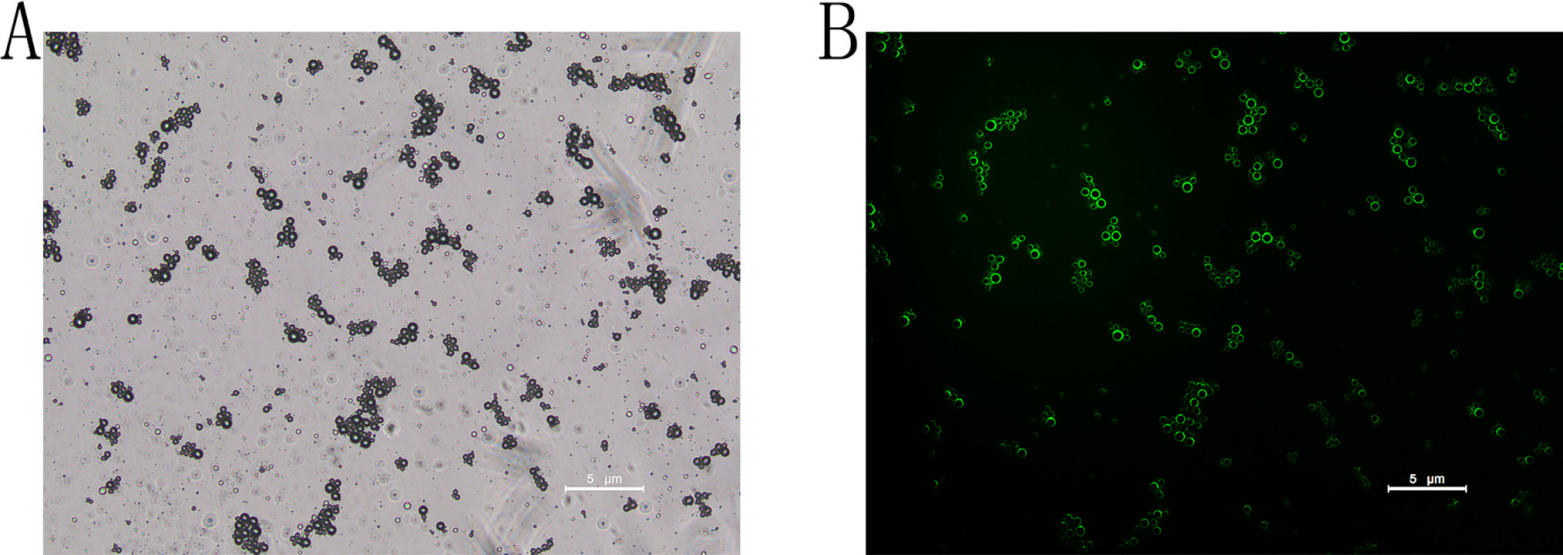
Visualization of FITC-labeled siRNA-SMBs complexes (**A**) The complexes showed uniform sizes, light density, and smooth round surfaces under a light microscope. (**B**) The complexes appeared as a pale green suspension; the surfaces appeared green under confocal laser scanning microscopy, indicating that FITC-labeled NET-1 siRNA was packaged on the surfaces of SMBs.

The effective diameters of pure sub-micron bubbles were 562.2 ± 30.9 nm, with a polydispersity value of 0.126 (Figure 1A). Next, we conjugated NET-1 siRNA and targeting ligand, the effective diameters of the NET-1 siRNA-SMBs complexes were 610.8 ± 26.3 nm (Figure 1B). Meanwhile, the zeta potential value of the complexes was –1.46 ± 3.14 mV (Figure 1C), because the siRNA conjugated on the surface is negatively charged. The NET-1 siRNA-SMBs complexes had a smooth round surface (Figure 2A). The FITC-labeled NET-1 siRNA emitted green fluorescence (Figure 2B).

The concentration of NET-1 siRNA was 335 ng/ul, and the concentration of siRNA-SMBs complexes was 3 × 10^8^/ml, so there was 1.12 × 10^–12^ g siRNA per one bubble particle. The average molecular weight of NET-1 siRNA was 13300, and the Avogadro’s constant was 6.02 × 10^23^, so the nucleic acid molecules of per one bubble particle were 5.97 × 10^7^. The result was calculated, while the bubbles were excess.

### Transfection efficiency of siRNA-SMB complexes combined with LFUS exposure

**Figure 3:**
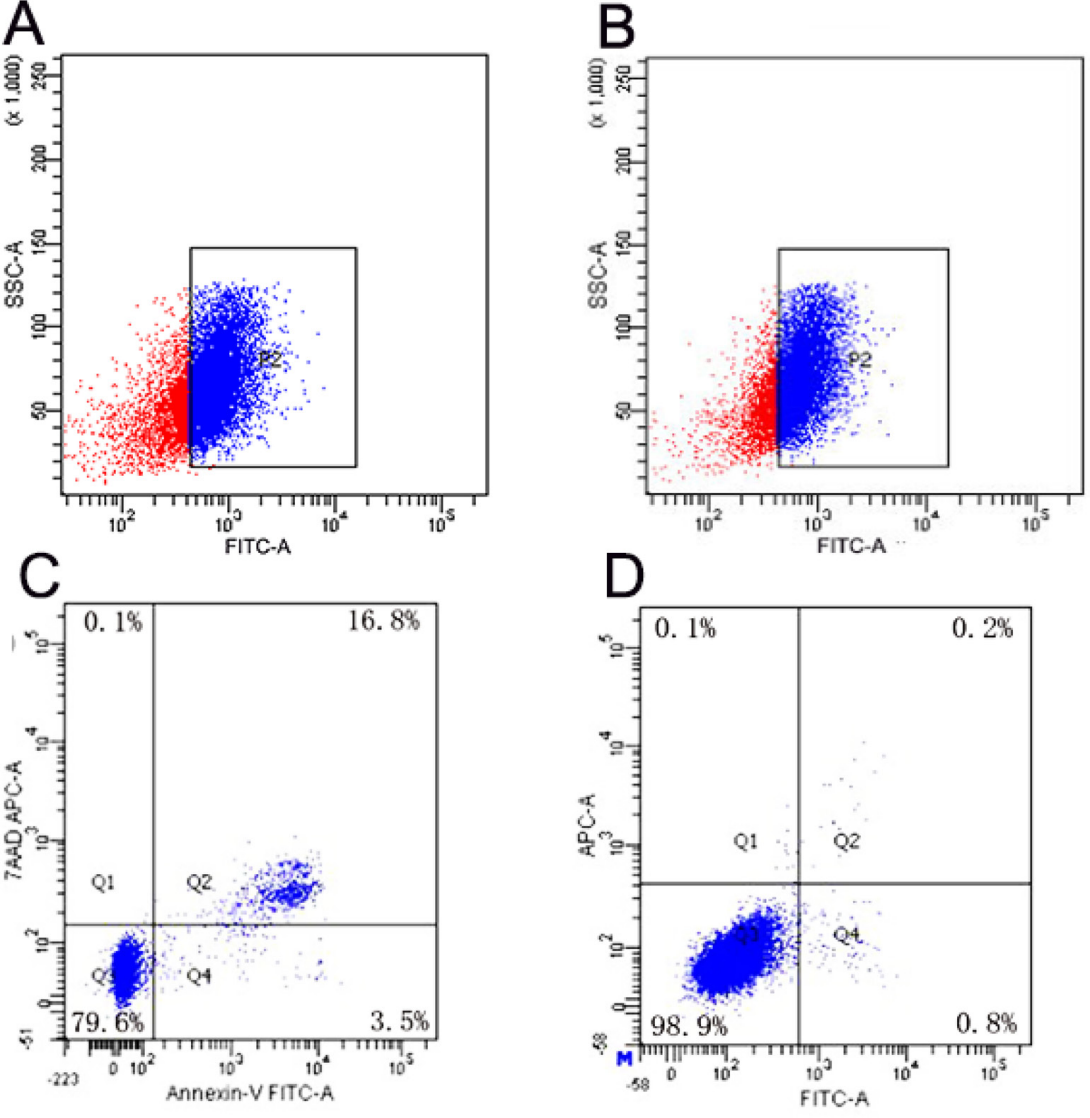
Results of transfection efficiency and apoptosis (**A**) Analysis of FITC-positive SMMC-7721 cells in group G: 79.77 ± 1.72% were FITC-positive, indicating highest transfection efficiency in group G. (**B**) Group F showed the second highest transfection efficiency: 64.32 ± 1.37% FITC-positive. (**C**) The typical result of apoptosis in group G. The analysis of apoptotic cells indicated that cells in group G manifested maximum apoptosis. (**D**) A representative of the result of apoptosis in group A. Almost no apoptosis was observed in the negative control group.

Flow cytometry revealed that the transfection efficiency of NET-1 siRNA-SMB complexes improved significantly when coupled with LFUS exposure. In group G, the suspension SMMC-7721 cells showed the highest transfection efficiency: 79.77 ± 1.72% cells were FITC-positive (Figure 3A), followed by group F that showed 64.32 ± 1.37% FITC-positive cells (Figure 3B). In contrast, cells in group A were all FITC-negative, then group B and C showed nearly no FITC-positive cells. The transfection efficiencies of group D and E were a little higher than group B and C, which were 21.9 ± 0.65% and 27.13 ± 1.48% (Supplementary Figure 1). There was no statistical significance between the two groups. The transfection efficiencies of group F and G were significantly higher than the other groups. Cell apoptosis assay indicated that the cells in group G exhibited maximum apoptosis, 13.83 ± 2.5% (Figure 3C), whereas, group A displayed almost no apoptosis (Figure 3D). It could be concluded that NET-1 siRNA-SMBs and LFUS irradiation could promote the siRNA transfection and induce the apoptosis observably.

The *in vivo* study was in progress, and the results of preliminary experiment with nude mice bearing tumor were exhibited in Supplementary Figures 2, 3 and Supplementary Video 1.

### Transwell assay

**Figure 4:**
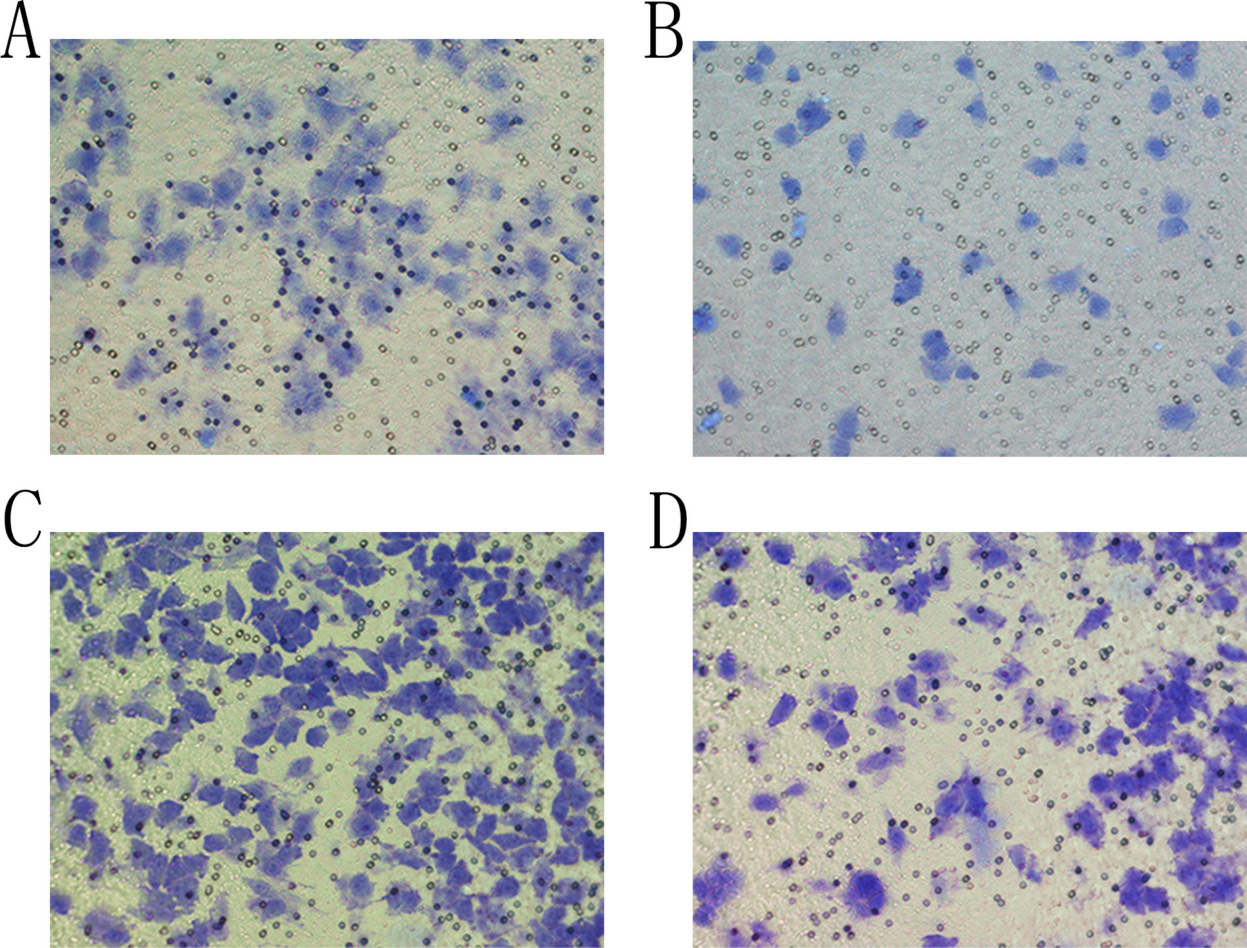
The result of Transwell assay (**A**) In a negative control group, SMMC-7721 cells were observed at the downside of the chamber. (**B**) Transfection with siRNA-SMB complexes combined with LFUS inhibited the migration ability of SMMC-7721 cells in group F. (**C**) SMMC-7721 cells were observed at the bottom of the BioCoat Matrigel invasion chamber in negative control group. (**D**) After transfection with siRNA-SMB complexes combined with LFUS, the number of the cells invading through the downside of the BioCoat Matrigel invasion chamber was decreased sharply.

The migration and invasion abilities of the cells were evaluated post-transfection. The group F showed that the number SMMC-7721 cells migrating towards the lower chamber reduced from 247.62 ± 23.41 to 65.28 ± 7.45 per field (*P* < 0.05; Figure 4A, 4B). The cells invading through the BioCoat Matrigel invasion chambers reduced from 452.41 ± 61.38 to 119.43 ± 41.27 per high field (*P* < 0.05; Figure 4C, 4D). This result indicated that NET-1 siRNA-SMB complexes and LFUS exposure significantly suppressed the cytoactivity.

### Silencing NET-1 gene expression

**Figure 5:**
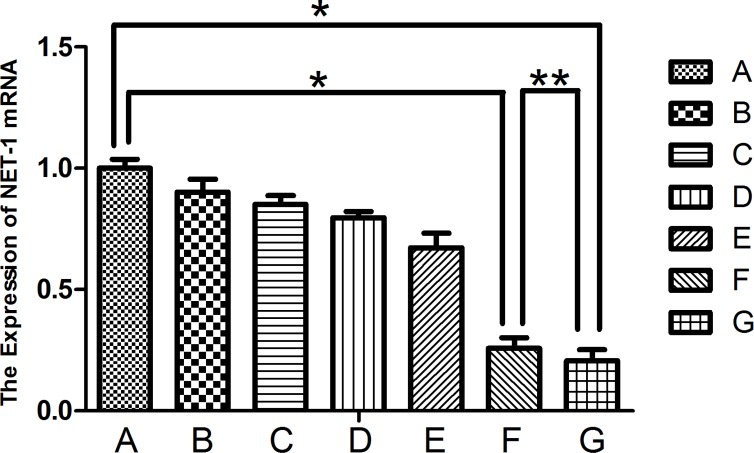
Expression of NET-1 mRNA Groups F and G showed a lower expression of NET-1 mRNA as compared to group A, ^*^P < 0.05. The expression between the two groups did not differ significantly (^**^P > 0.05).

The result of NET-1 siRNA concentration was showed in Supplementary Figure 4. The *NET-1* gene expression was silenced rather efficiently in groups F and G that inhibited the expression of NET-1 mRNA than that in other groups (*P* < 0.05; Figure 5). The expression of mRNA was markedly reduced in groups F and G as compared to group A (*P* < 0.001). Furthermore, group F showed a 74.3 ± 4.5% decrease, and group G showed a 79.3 ± 4.4 % decrease in the NET-1 mRNA level. The expression between the two groups did not differ significantly (^**^P = 0.1755).

**Figure 6:**
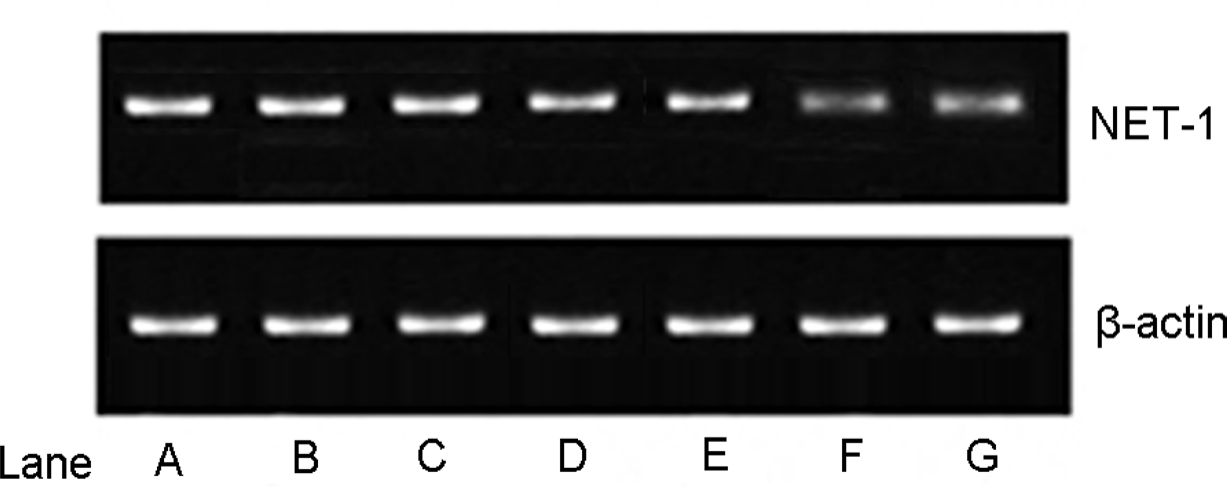
Expression of the NET-1 protein Lanes A–F represented groups A–F, β-actin served as an internal reference. The expression of the NET-1 protein of groups F and group G was lower than any of the other groups, all *P* < 0.05. However, no difference was observed between groups F and G by the naked eye.

The expression of the NET-1 protein was suppressed distinctly as observed in groups F and G (Figure 6), which was in agreement with the results by qRT-PCR. However, no difference in the protein expression was visible to the naked eye.

The expression of the NET-1 protein in groups F and G was significantly lower than the other groups (both *P* < 0.01). Compared to the negative control group, the inhibition ratio of the protein expression was 95.41 ± 3.32% and 94.63 ± 2.24% in groups F and G, respectively. However, no significant differences were not observed between the two groups (*P* = 0.5551).

These results proved that NET-1 siRNA-SMB complexes and LFUS exposure could deliver NET-1 siRNA to SMMC-7721 cells, exhibiting high-performance and inhibit the NET-1 mRNA and protein expressions.

## DISCUSSION

In the present study, we investigated the function of NET-1 siRNA-SMB complexes combined with LFUS in gene transfection. We could confirm that the NET-1 mRNA was highly expressed in HCC cell lines and that the protein was expressed mainly in the cytoplasm of the cells. The inhibition of NET-1 mRNA expression in HCC cell lines using NET-1 siRNA-conjugated SMBs combined with LFUS decreased not only the proliferation but also the migration and invasiveness as substantiated in the present study. These findings suggested that NET-1 gene contributes to the pathogenesis of HCC. To improve the efficiency of NET-1 siRNA delivery, we used the NET-1 siRNA-SMBs complexes combined with LFUS to deliver NET-1 siRNA in adherent cells and suspension cells. We speculate that the siRNA-SMB complexes might effectively potentiate the siRNA transfection *in vitro*. When the cells were subjected to the LFUS exposure, cavitations appeared owing to the presence of siRNA-SMBs, resulting in permeability enhancement in cancer cell membranes [23, 24]; the endocytosis was also enhanced. Therefore, siRNA was effectively delivered into tumor cells. In addition, the siRNA conjugated to the SMBs was released into the tumor cells upon cavitation and further driven into the cytoplasm by acoustic streaming that exhibited a sonoporation effect on the cancer parenchymal cell membrane [25]. On the other hand, SMBs could also protect the siRNA from degradation by RNase [26].

The effective size of the NET-1 siRNA-SMB complexes was a critical factor in gene transfection. The complexes should be sufficiently small in order to be able to penetrate the interval of vascular endothelial cells and deliver the siRNA into the cells. The pore size of the vasculature was between 380 and 780 nm [27, 28].

The effective size of NET-1 siRNA-SMB complexes, constructed in our laboratory, was 610.8 ± 26.3 nm as shown in Figure 1. Compared to the conventional microbubbles, the SMBs could deliver excessive siRNA into cells. The NET-1 siRNA-SMB complexes had smooth round surfaces and uniform sizes as shown in Figure 2A. The confocal laser scanning microscopy demonstrated the FITC-labeled siRNA conjugated equally around the surfaces of SMBs emitted green fluorescence (Figure 2B). Taken together, Figures 1 and 2, indicated that the NET-1 siRNA-SMB complexes had been synthesized successfully.

The flow cytometry data showed that the NET-1 siRNA-SMB complex-based delivery of siRNA resulted in substantial gene transfection efficiency in both adherent and suspension cells than any other groups in Figure 3A, 3B. This approach significantly reduced the expression of NET-1 mRNA, causing cellular apoptosis as shown in Figure 3C. Contrastingly, we hardly observed any apoptosis in the negative control group (Figure 3D).

In addition, the migration and invasion of SMMC-7721 cells were assessed by the Transwell assay. A minimum number of cells in group F migrated and invaded through the lower chamber of the Transwell assembly. However, the result could be inaccurate as it was contradictory to that observed by flow cytometry and molecular biology assays. Therefore, we concluded that the cells in group F were adherent growth before transfection. Before the Transwell assay, the cells in group F were harvested using 0.25% trypsin–EDTA, which might have exerted an inhibitory effect on the cytoactivity, thereby impeding the cells’ penetration in the Transwell chamber. Under similar conditions as group G, the cells in group F showed worse migration and invasion abilities. Consequently, there were only a few cells in the lower chamber of the Transwell assembly, in group F.

Nevertheless, the cytoactivity of the cells in groups F and G weakened remarkably than the negative control group A, suggesting that NET-1 siRNA-SMB complexes efficiently inhibited the HCC metastasis.

The results of the molecular biology assays indicated that the NET-1 siRNA-SMB complexes combined with LFUS exposure could deliver NET-1 siRNA into the cells efficiently, leading to the inhibition of NET-1 mRNA expression and protein. Notably, the NET-1 mRNA expression levels in groups F and G were lower than the negative control group A (Figure 5; ^*^P < 0.001). However, the group F did not differ significantly from group G (^**^P = 0.1755). Moreover, a subtle difference was observed between groups F and G (Figure 5); the NET-1 mRNA expression inhibition ratios of groups F and G were 74.3 ± 4.5% and 79.3 ± 4.4%, respectively. The statistical analysis did not reveal any significance, thereby indicating the lack of considerable difference in transfection efficiency between adherent and suspension cells.

According to the Western blot results (Figure 6), the NET-1 protein expression in groups F and group G was lower than any other groups, which corroborated with the results by the qPCR assay. However, there a significant difference was not observed between the two groups (*P* = 0.5551). Nevertheless, the analysis of the Western blot result by Image J revealed a higher protein expression in group F than group G.

## CONCLUSIONS

A distinct difference in the transfection efficiency between adherent and suspension cells was not apparent as assessed by a series of experiments. Nevertheless, the NET-1 siRNA-SMB complexes served as gene carriers, bearing a gas core. With the aid of LFUS exposure, the complexes could release siRNA and deliver them into tumor cells efficiently.

The combination of LFUS exposure and SMBs for gene delivery exhibits some advantages, such as low cytotoxicity, reproducibility, and low immunogenicity [29, 30]. Therefore, a siRNA-SMB complex was a valuable advancement in gene delivery strategy as a potential clinical application. However, there are still some limitations of the siRNA-SMB complexes and LFUS-mediated gene therapy in cancers [31, 32]. Further studies are essential in order to ensure the safety and validity of the *in vivo* approach. Therefore, we aspire to explore the functions of siRNA-SMB complexes in nude mice and attempt to endue their targeting ability.

## MATERIALS AND METHODS

### NET-1 siRNA construction

We designed the NET-1 siRNA duplex sequences according to the published NET-1 sequence from the GenBank database (accession no. AF065388), using Qiagen siRNA software (Qiagen, Shanghai, China). The NET-1 siRNA duplex and negative control sequences were synthesized and NET-1 siRNA duplex was modified by biotin and FITC labels, respectively (Genpharm, Shanghai, China). The sequence was shown in Table 1.

**Table 1:** Sequence of NET-1 siRNA duplex and negative control

Gene	siRNA Duplex
NET-1 siRNA	Sense: 5′-GGG CAU CCU UUC UGA AGA UTT-3′
	Antisense: 5′-AUC UUC AGA AAG GAU GCC CTT -3′
Negative control	Sense: 5′-UUC UCC GAA CGU GUC ACG UTT-3′
	Antisense: 5′-ACG UGA CAC GUU CGG AGA ATT-3′

### Sub-micron bubbles preparation

The SMBs were formulated with 1,2-distearoyl-sn-glycero-3-phosphoethanolamine (DSPE; MW: 748.06, Avanti Polar Lipids Inc., USA), 1,2-distearoyl-sn-glycero-3-phosphocholine (DSPC; MW: 790.145, Avanti Polar Lipids), and 1,2-distearoyl-*sn*-glycero-3-phosphoethanolamine-N-[biotinyl(polyethyleneglycol)-2000] (DSPE-PEG2000-biotin, MW: 3016.781, Avanti Polar Lipids) at molar ratios of 18:1:1. 20mg of the phosphatide was solubilized in a mixture of chloroform and methanol and evaporated at 37°C. The phosphate buffered saline (PBS) was used to hydrate the dried phospholipid mixture in sealed vials. Then, the air in the vial was replaced by perfluoropropane (C_3_F_8_; Research Institute of Physical and Chemical Engineering of Nuclear Industry, Tianjin, China). Next, the admixture was mechanically vibrated for 45 s in a dental amalgamator, and then a 20-kHz probe (Sonics, USA) was used to sonicate the admixture for 20 s. Finally, the blend was resuspended in 5 mL PBS and centrifuged at 200 × *g* for 5 min. The supernatant collected was SMBs and was estimated by a hemocytometer.

### Fabrication and characterization of NET-1 siRNA-SMB complexes

The SMBs were conjugated with 400 µL avidin (10 mg/mL, Sigma, USA) by incubation at 25°C. Subsequently, the SMBs were washed with PBS at 200 × *g* and resuspended in PBS. The avidinylated SMBs and biotinylated NET-1 siRNA were gently agitated and stored overnight at 4°C in order to allow the formation of the siRNA-SMB complexes. Then, the complexes were washed three times with PBS. The particle size and Zeta potential were measured by t a Zetasizer 3000HS (Malvern Zetasizer Nano ZS; USA). The complexes were observed by confocal laser scanning microscopy (Olympus, Japan).

The nucleic acid molecules amount of NET-1 siRNA conjugated to siRNA-SMBs complexes was determined by the absorbance of 260 nm using Gene Quant pro (Amersham Biosciences, UK).

### Cell culture

SMMC-7721 cells, a human HCC cell line, were used in the transfection. The cells were cultured in DMEM medium (Invitrogen, USA) containing 10% fetal bovine serum (FBS, Gibco, USA) in a 5% CO_2_ incubator at 37°C. Routine passages were performed with trypsin digestion.

### Gene transfection combined with LFUS exposure *in vitro*

The cells were divided into 7 treatment groups as shown in Table 2. Before treatment with LFUS exposure, SMMC-7721 cells were seeded in 6-well plates (Costar Corp, USA), at a confluency of 50% per well.

**Table 2:** Groups of experiments and transfection treatment

Groups	Treatment	Abbreviation
**A**	negative control and LFUS	NC + LFUS
**B**	adherent cells and NET-1 siRNA-SMBs	AC + siRNA-SMBs
**C**	suspension cells and NET-1 siRNA-SMBs	SC + siRNA-SMBs
**D**	adherent cells and Bare NET-1 siRNA + LFUS	AC + siRNA + LFUS
**E**	suspension cells and Bare NET-1 siRNA + LFUS	SC + siRNA + LFUS
**F**	adherent cells and NET-1 siRNA-SMBs + LFUS	AC + siRNA-SMBs + LFUS
**G**	suspension cells and NET-1 siRNA-SMBs + LFUS	SC + siRNA-SMBs + LFUS

In Group A, B, D and F, the adherent cells were cultured in 5 ml medium containing 10% FBS overnight. Before transfection, the medium in each well was substituted by 4 ml of DMEM medium without FBS. Next, added 1 ml negative control duplex into Group A and exposed the cells with LFUS, added 1ml siRNA-SMBs complexes into Group B without LFUS irradiation, added 1 ml bare NET-1 siRNA duplex into Group D and exposed the cells with LFUS, added 1ml NET-1 siRNA-SMBs complexes into Group F and exposed the cells with LFUS.

In Group C, E, and G, the suspension cells were digested with 0.25% trypsin–EDTA (Hyclone, USA) and washed two times with PBS before transfection followed by resuspended in 4 mL DMEM medium without FBS. Next, added 1ml NET-1 siRNA-SMBs complexes into Group C without LFUS irradiation, added 1ml bare NET-1 siRNA duplex into Group E and exposed the cells with LFUS, added 1ml NET-1 siRNA-SMBs complexes into Group G and exposed the cells with LFUS.

The concentration of NET-1 siRNA-SMBs complexes was 3 × 10^8^/ml. The LFUS transfection apparatus (Institute of Ultrasound Imaging, Second Affiliated Hospital of Chongqing Medical University, Chongqing, China) parameters: power density of 1.0 W/cm^2^, duty cycle of 20%, and the frequency were 1 MHz, and the LFUS irradiation time was 60 s.

### Transfection efficiency and apoptosis analysis

After transfection for 24 h, the adherent SMMC-7721 cells were washed two times with ice-cold PBS to remove the dead cells and harvested with 0.25% trypsin–EDTA. Then, the cells were resuspended in binding buffer at a concentration of 1 × 10^6^ cells/mL, followed by transfer of 100 µL of the solution (1 × 10^5^ cells) to a 5 mL culture tube. Subsequently, 5 µL FITC Annexin V and 5 µL 7-AAD were added to the tube and incubated at 25°C in the dark for 15 min. Finally, 400 µL binding buffer was added to each tube, and the fluorescence intensity of the cells was measured by flow cytometry (BD Biosciences, USA), and the results were analyzed by NovoCyte flow cytometer (ACEA Biosciences, USA) [33].

### Transwell assay

The cell migration and invasion were assessed using Cell Culture Inserts with 8.0-μm pore membranes in the Transwell chambers (Costar Corp., USA) that were placed in a 24-well plate (Costar Corp.).

For the migration assay, 1 × 10^4^ cells were seeded on the upper chamber in 100 μL medium containing 1% FBS, and 800 μL medium containing 10% FBS was placed in the lower chamber. The cells were allowed to migrate to the underside of the membrane for 12 h, following which, the membrane in the lower chamber was stained with 0.5% crystal violet (Sigma, USA).

In order to evaluate the invasion ability, the BioCoat Matrigel invasion chambers (BD Biosciences, USA) were utilized, and 5 × 10^4^ cells suspended in 200 μL DMEM containing 1% FBS were seeded in the pre-coated chamber. After 24 h incubation in 5% CO_2_ incubator at 37°C, the cells were stained as described above.

The cells passed that through the chambers were quantified by counting three fields (magnification, 200).

### Quantitative real-time PCR (qPCR)

The mRNA levels of *NET-1* were determined using qPCR. SMMC-7721 cells were seeded in 6-well plates at a density of 3 × 10^5^ cells/well, and cultured overnight in 5 mL growth medium containing 10% FBS. The cells were treated as described previously. After transfection for 24 h, the total RNA was isolated from cells using the High Pure RNA Isolation Kit (Roche Diagnostics, Germany) and transcribed into first-strand cDNA using a Transcriptor First Strand cDNA Synthesis Kit (Roche Diagnostics). A 20 μL PCR reaction mixture contained the forward and reverse primers, hydrolysis probe, FastStart Universal Probe Master reagent (ROX, Roche Diagnostics), and 2 μL cDNA. Glyceraldehyde-3-phosphate dehydrogenase (*GAPDH*) was quantified in each sample as an endogenous control. The primers sequence of *NET-1* and *GAPDH* were shown in Table 3. The reverse transcription was performed as follows: 25°C for 10 min and 50°C for 60 min. ABI 7500 FAST Real-Time PCR System (Applied Biosystems Inc., USA) was utilized to perform the qPCR according to the following conditions: activation of FastStart Taq DNA Polymerase at 95°C for 10 min, then 50 cycles at 95°C for 10 s, and 60°C for 30 s. The expression level of each sample was evaluated in triplicate, and the data was quantified using the 2^–ΔΔCT^ method.

**Table 3:** Sequence of NET-1 and GAPDH primers

Primer	Sequence	
**NET-1**	5′-AGTGCCTTTCCCCCATTCTG-3′	(forward)
	5′-TCGTGAGCCTTTTGCTTGGT-3′	(reverse)
	5′-TGCAATGACAACGTCACCAACACAGC-3′	(probe)
**GAPDH**	5′-TGGAAGGACTCATGACCACAGT-3′	(forward)
	5′-GCCATCACGCCACAGTTTC-3′	(reverse)
	5′-CATGCCATCACTGCCACCCAGAAGA-3′	(probe)

### Western blot analysis

Cells were harvested 24 h post-transfection using the LFUS exposure method described above and subjected to Western blot analysis as reported previously [34]. The cells were washed two times with cold PBS and suspended in cell lysis buffer to extract the NET-1 protein. The protein concentration was measured using Pierce BCA Protein Assay Kit (Thermo Scientific, USA) according to the manufacturer’s protocol. An equivalent of 50 µg cell lysate was resolved by SDS-PAGE and transferred to a polyvinylidene fluoride (PVDF) membrane. The membrane was blocked using 5% nonfat milk powder in Tris-buffered saline with 0.05% Tween-20 (TBST) for 4 h at room temperature and probed with the specified primary antibody (ab55484, mouse monoclonal antibody against human AR; Abcam, Cambridge, UK; 1:400 dilution) overnight at 4°C. Then, the membrane was washed with TBST, followed by incubation with a secondary antibody (ab6789, goat anti-mouse IgG; Abcam; 1:1200) at 37°C for 2 h. The specific protein band signals were visualized using an enhanced chemiluminescence system (ECL; Amersham Pharmacia, Roosendaal, Netherlands), and analyzed by Image J software (1.46r, Wayne Rasband, National Institutes of Health, USA). β-actin was adopted as an endogenous control [35].

### Statistics and analyses

All data were expressed as the mean ± standard deviation of triplicate experiments. The data were analyzed using unpaired *t*-test with Welch’s correction (Prism 6.0 software, GraphPad Software, La Jolla, CA, USA,). A *P*-value < 0.05 was regarded as statistically significant.

## SUPPLEMENTARY MATERIALS FIGURES AND VIDEO




