# Supplementary material for: Effect of NET-1 siRNA conjugated sub-micron bubble complex combined with low-frequency ultrasound exposure in gene transfection
Source: Oncotarget. 2017 Dec 23;9(3):4150–60. doi: 10.18632/oncotarget.23646 (PMC5790528; doi:10.18632/oncotarget.23646)
Supplement: Supplementary file 1 [file oncotarget-09-4150-s001.pdf]

Effect of NET-1 siRNA conjugated sub-micron bubble complex combined with low-frequency ultrasound exposure in gene transfection

SUPPLEMENTARY MATERIALS

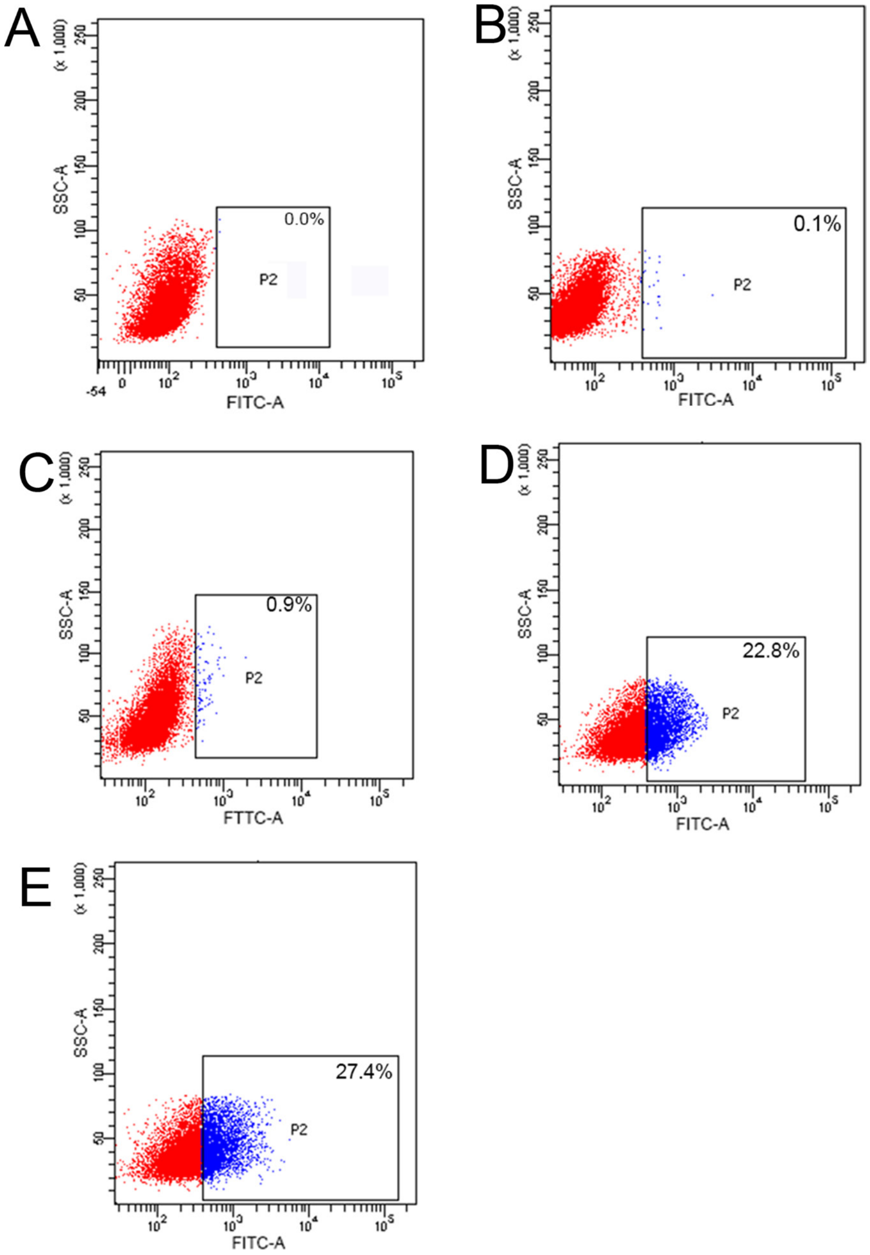

Supplementary Figure 1: The transfection efficiencies of other groups. (**A–C**) There was nearly no FITC-positive cells in group A, B and C; (**D**) Group D showed 21.9 ± 0.65% FITC-positive cells; (**E**) Group E showed 27.13 ± 1.48% FITC-positive cells.

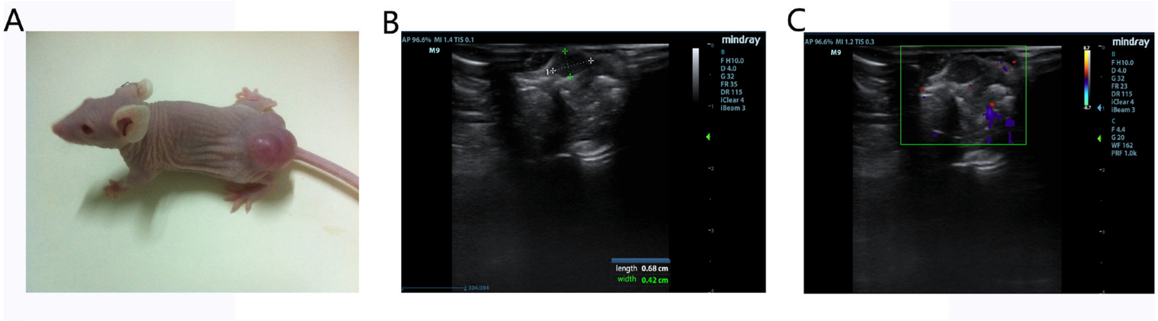

Supplementary Figure 2: The figure of *in vivo* study. (**A**) The ordinary image of BALB/c nude mice bearing tumor; (**B**) The image of two dimensional ultrasonography; (**C**) The image of two dimensional Color Doppler flow imaging (CDFI).

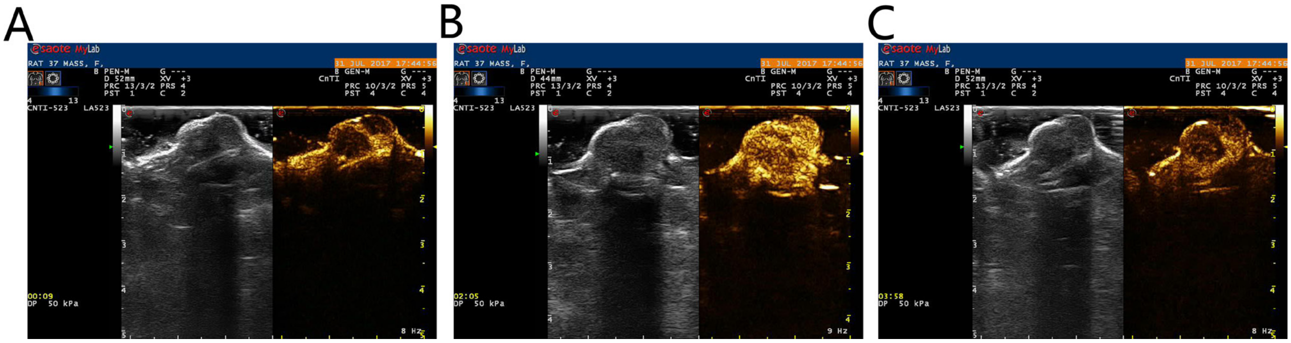

Supplementary Figure 3: The figure of contrast-enhanced ultrasound *in vivo*. (**A**) The tumor image before injecting siRNA-SMB complexes; (**B**) The enhanced tumor image after injecting 125 s, the tumor was filled with siRNA-SMB complexes; (**C**) After 4 min, there was no enhancement in the tumor.

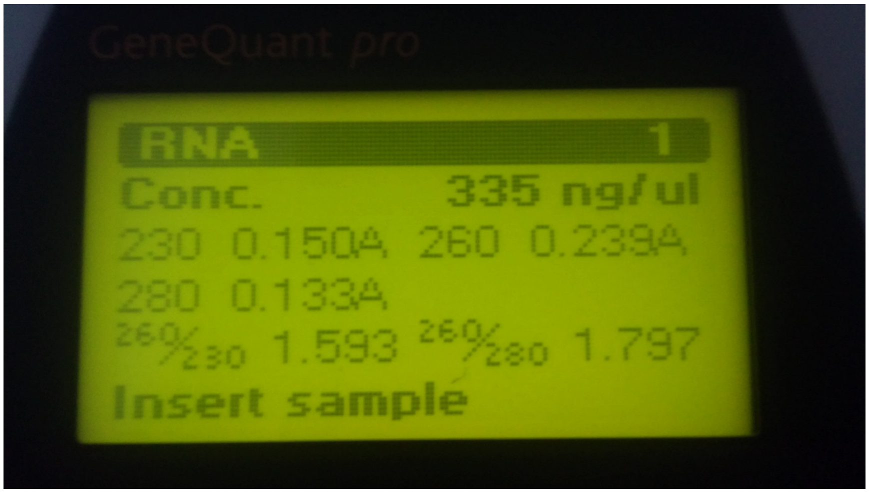

Supplementary Figure 4: The result of NET-1 siRNA concentration. The siRNA concentration was determined by the absorbance of 260 nm using Gene Quant pro.

Supplementary Video 1: The real-time record of contrast enhanced ultrasound with siRNA-SMB complexes. See Supplementary_Video_1
